# The Mitochondrion-Located Protein OsB12D1 Enhances Flooding Tolerance during Seed Germination and Early Seedling Growth in Rice

**DOI:** 10.3390/ijms150813461

**Published:** 2014-07-31

**Authors:** Dongli He, Hui Zhang, Pingfang Yang

**Affiliations:** 1 Key Laboratory of Plant Germplasm Enhancement and Specialty Agriculture, Wuhan Botanical Garden, Chinese Academy of Sciences, Wuhan 430074, China; E-Mails: hedongli@wbgcas.cn (D.H.); zhanghui@wbgcas.cn (H.Z.); 2 Graduate University of Chinese Academy of Sciences, Beijing 100049, China

**Keywords:** OsB12D1, flooding tolerance, mitochondrion, rice

## Abstract

B12D belongs to a function unknown subgroup of the *Balem* (Barley aleurone and embryo) proteins. In our previous work on rice seed germination, we identified a B12D-like protein encoded by LOC_Os7g41350 (named *OsB12D1*). OsB12D1 pertains to an ancient protein family with an amino acid sequence highly conserved from moss to angiosperms. Among the six *OsB12D*s, *OsB12D1* is one of the major transcripts and is primarily expressed in germinating seed and root. Bioinformatics analyses indicated that *OsB12D1* is an anoxic or submergence resistance-related gene. RT-PCR results showed *OsB12D1* is induced remarkably in the coleoptiles or roots by flooding during seed germination and early seedling growth. The *OsB12D1*-overexpressed rice seeds could protrude radicles in 8 cm deep water, further exhibiting significant flooding tolerance compared to the wild type. Moreover, this tolerance was not affected by the gibberellin biosynthesis inhibitor paclobutrazol. OsB12D1 was identified in the mitochondrion by subcellular localization analysis and possibly enhances electron transport through mediating Fe and oxygen availability under flooded conditions. This work indicated that *OsB12D1* is a promising gene that can help to enhance rice seedling establishment in farming practices, especially for direct seeding.

## 1. Introduction

In orthodox seeds, metabolic activity is interrupted during desiccation of maturation and achieves a physiologically relatively quiescent status in which the embryonic cell viability can extend over centuries [1]. Following simple imbibition, the non-dormant seeds will initiate the germination process, restart metabolic activity, mobilize the reserves, biosynthesize new proteins, regenerate organelles and cell membranes, eventually protrude the radicle and enter into seedling establishment [2].

Seed germination is a complex regulation system. Numerous exogenous and endogenous factors can affect the germination process, including seed vigor, water, temperature, oxygen and phytohormones [3,4]. Exploration of the functional genes that respond to those factors is an ongoing routine work for seed biologists to elucidate the complex molecular mechanisms underlying seed germination. Forward and reverse genetic methods have been adopted to scan the functional genes. Using retrotransposon Tos17 as an endogenous insertion mutagen, the *Osaba1* gene that controls seed viviparous by improving ABA levels was cloned in rice [5]. Bentsink *et al.* had cloned the quantitative trait locus controlling dormancy (*DOG1*) in *Arabidopsis* [6]. Recently, through screening the rice T-DNA insertion mutant library, a *gd1* mutant that is characterized by seed germination defects and reduction of GA biosynthesis was obtained [7]. Various developed “-omics” approaches have greatly expanded the investigation on the mechanism of seed germination [8,9,10,11]. A series of genes that manifest vital functions during seed germination are identified, especially for gene response to gibberellin (GA) and abscisic acid (ABA), two antagonistic phytohormones regulating seed germination [12]. In farming conditions, environmental stresses constrain crop seed germination; seeds have established the intensive molecular mechanism to resist those stresses. For example, protein RAP2.6, an *Arabidopsis* AP2/ERF family member, can function in multiple abiotic stresses during seed germination, including salt, osmotic, and cold stress [13]. Additionally, multiple functional genes can play roles in orchestra to resist stresses. Direct seeding of rice seeds in paddy fields will subject seeds to waterlogged or flooding stress which will expose seeds to hypoxia or even anoxia [14]. Under hypoxia, α-amylase (*Amy1A* and *Amy3D*) and sucrose synthase (*Sus3*) genes were activated to degrade starch and sucrose, pyruvate decarboxylase (*Pdc1* and *Pdc2*) and alcohol dehydrogenase (*Adh1* and *Adh2*) genes were induced to adjust the low-oxygen stress by enhanced ethanol fermentation, and ethylene biosynthesis-related genes were greatly increased and then initiated a cascade of reactions to promote the coleoptile elongation [15,16,17,18].

As the alive tissues in the cereal dry seed, the aleurone layer and the embryo share several characteristics, including synthesis and accumulation of lipid bodies and special protein bodies, desiccation tolerance, dormancy, and response to GA by inducing synthesis of hydrolytic enzymes to degrade the storage and promote seed germination [19]. The characteristic transcripts of aleurone and embryo have been investigated by differential screening of a cDNA library from barley alleurone [20], two main groups of clones, *Bal* (Barley aleurone) and *Balem* (Barley aleurone and embryo) were screened. In the *Balem* group, one subgroup of genes, including *Per1*, *Ole1* and *Ole2* having a *Lea*-like expression pattern, were up-regulated by ABA and mannitol and contributed to the desiccation tolerance and dormancy maintenance of mature seeds [19,21]. However, another subgroup, including B12D and B22E, had the opposite expression and stress response patterns. B12D and B22E were preferentially expressed in the aleurone layer and embryo of developing seeds and germinating seeds while disappearing in mature seeds [19,22,23]. Both of them were inhibited by ABA and mannitol in germinating seeds, but were differentially responsive to GA_3_: B12D could be induced by GA_3_ while B22E was insensitive to GA_3_. The function of the second subgroup remains unknown.

In our previous work on proteomics analysis of rice seed germination [11], we identified a B12D-like protein encoded by LOC_Os7g41350 (named *OsB12D1*). In the present study, we systematically analyzed the gene evolution, structure, expressional patterns, function, and protein subcellular localization of *OsB12D1*. Our data demonstrated that *OsB12D1* is a functional gene to enhance rice seedling establishment under flooded condition.

## 2. Results

### 2.1. OsB12D1 Pertains to an Ancient Protein Family

Using the full-length sequence of OsB12D1 protein sequence as the query to search against NCBI (The National Center for Biotechnology Information) database, 55 close homologs were obtained from 30 different species in addition to rice, belonging to moss, gymnosperm to angiosperms (Supplemental Dataset 1). All of these homologs were low-molecular proteins with the number of amino acids ranging from 76 to 134. Transmembrane region analysis using TMHMM software showed that each protein has a hydrophobic *N*-terminal [24], which indicated B12Ds might be membrane-anchored proteins.

**Figure 1:**
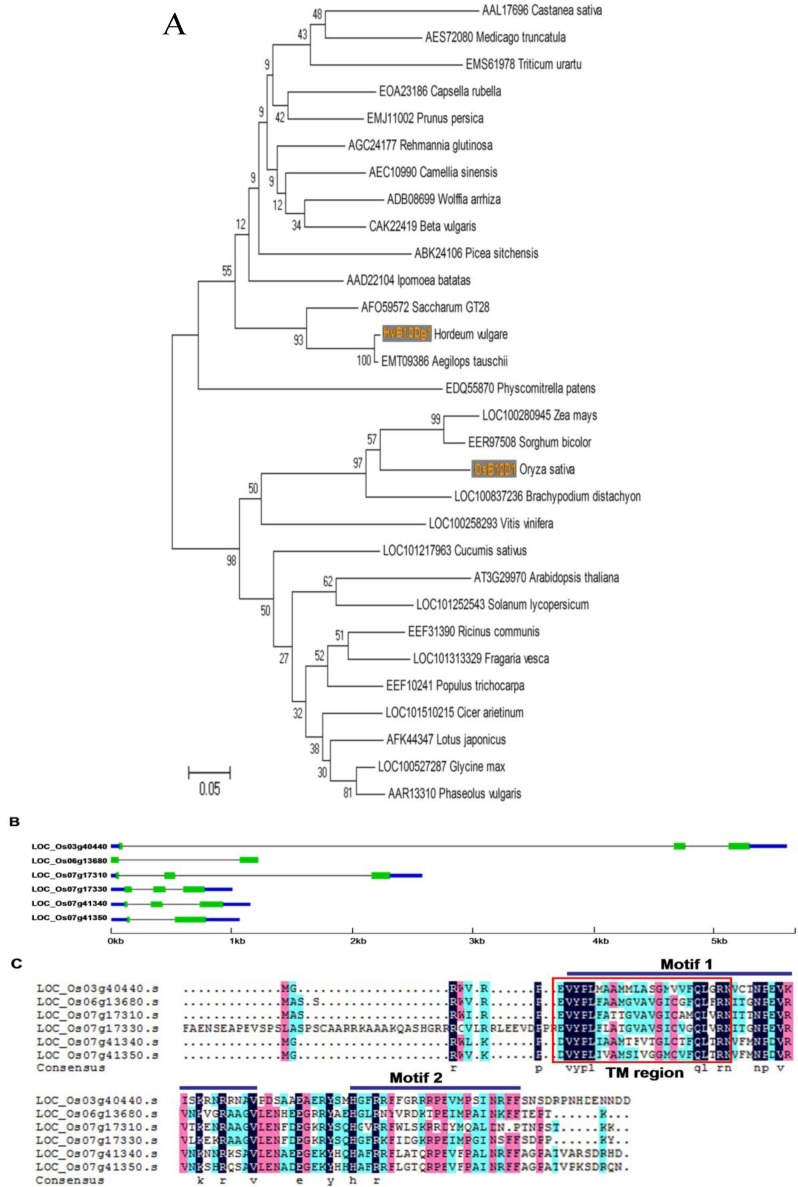
Sequence analyses of *OsB12Ds* (**A**) Phylogenetic analysis of OsB12D1 protein using neighbor-joining method with MEGA 4; bootstrap values from 1000 replicates are indicated at each node; the scale bar represents 5% sequence divergence. Those proteins were divided into two distinct clades, OsB12D1 and HvB12Dg1 belonged to different clades, respectively; (**B**) Gene structures of six *OsB12Ds*. green line, exons; gray line, introns; blue line, 3' or 5' un-translated region; (**C**) Multiple sequence alignment and transmembrane region of OsB12Ds proteins. Identical, conservative (5/6–1) and block (3/6–4/6) of similar amino acid residues are shaded in deep blue, cherry red and blue, respectively. The transmembrane regions and conserved motifs were marked by red rectangle and blue lines, respectively.

OsB12D1 and its 29 closest homologs from the other species were used for phylogenetic analysis with the neighbor-joining method (Figure 1A). All these B12D proteins were significantly clustered into two clades. Both clades consisted of B12Ds from monocotyledons and dicotyledons, which indicated that the main characteristics of the B12D family formed before the genetic split of monocotyledons and dicotyledons in plants. OsB12D1 was closely grouped with monocot counterpart proteins from brachypodium, maize and sorghum, while it diverged from the HvB12Dg1, sharing only 46% identity.

### 2.2. Gene Structure and Expressional Patterns of OsB12Ds

From the RGAP (Rice Genome Annotation Project) database, six OsB12D paralogs were obtained, including two pairs of tandem duplicates (LOC_Os07g17310 and LOC_Os07g17330; LOC_Os07g41340 and LOC_Os07g41350). Gene structure analysis showed that all *OsB12D* genes harbored only two or three exons, and although they have similar CDS size, the intron fragments’ size varied greatly (Figure 1B). The six OsB12Ds proteins shared two conserved motifs, including the N-terminal transmembrane region (Figure 1C).

**Figure 2:**
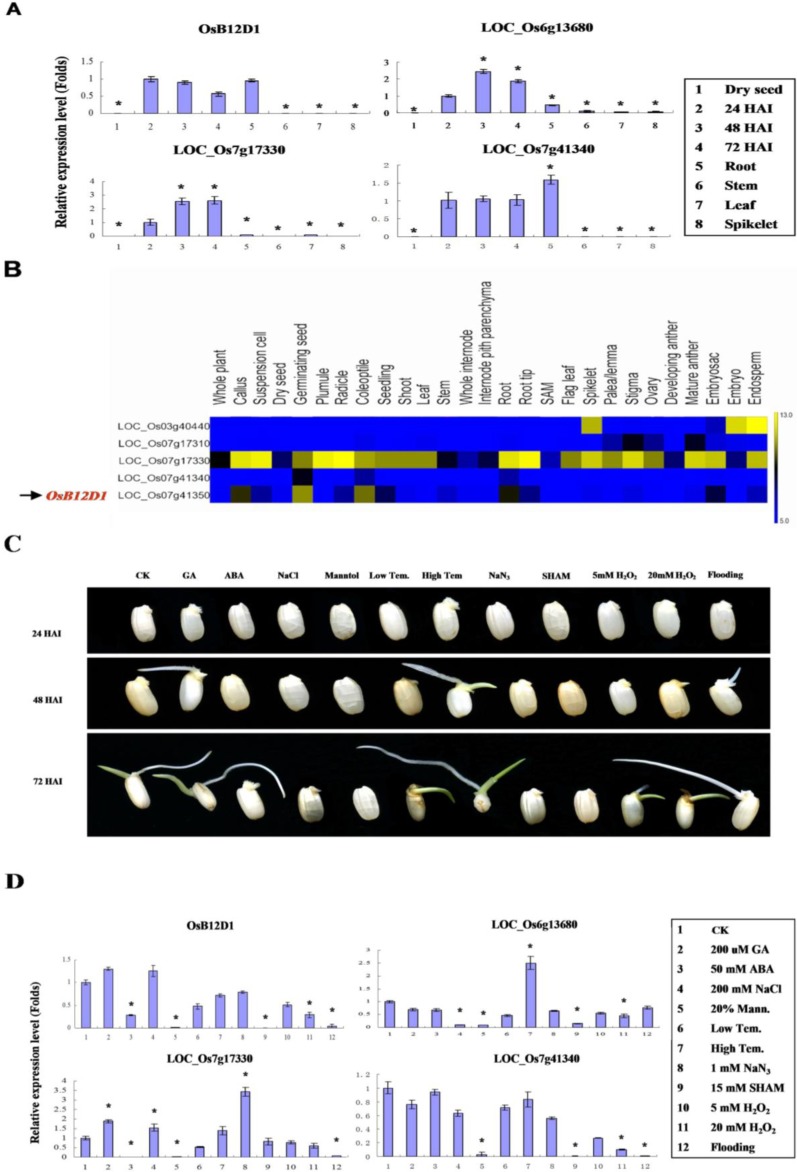
Expression analyses of *OsB12Ds*. (**A**) qRT-PCR analysis of the *OsB12Ds* expressional pattern in different stages of rice seed germination and various tissues. The RNA was extracted from embryos of the dehulled rice seeds at 0, 24, 48, and 72 h after imbibition (HAI) under normal condition and tissues of root, stem, leaf and spikelet of 60 day-old rice plant; (**B**) Digital expression patterns of *OsB12Ds.* Five OsB12Ds were obtained from the ricearray database using meta-analysis tool [25]; (**C**) Rice seeds germinating under multiple treatments for 24 h. From left to right: CK, control; GA, 200 µM GA3; ABA, 50 mM ABA; NaCl, 200 mM NaCl; Mannitol, 20% Mannitol; Low Tem, seeds were imbibed at 4 °C for three days in the dark before being transferred to germinate under benign condition; High Tem, seeds pretreated at 40 °C and 100% relative humidity for three days; NaN_3_, 1 mM NaN_3_; SHAM, 15 mM SHAM; 5 mM H_2_O_2_; 20 mM H_2_O_2_; Flooding, seeds were directly submerged in 8 cm deep of distilled water; (**D**) qRT-PCR analysis of the *OsB12D* expression under multiple treatments. The embryos of 24 HAI rice seeds under multiple treatments were excised manually and used for RNA extraction. The expression of *OsB12D1* was normalized to the expression of rice 18 S rRNA. The values are the means with the standard deviations (*n* = 3), asterisks (* *p* < 0.01, Student’s *t*-test) represent significant differences from the expression level in 24 HAI seed embryo under normal conditions.

Quantitative RT-PCR method was used to examine the expression pattern of the *OsB12Ds* in the embryo of germinating seed at different stages and various tissues from a 60-day-old rice plant. Four *OsB12Ds* were detected in most tissues and had a relatively high expression level in germination seed but low in dry seeds, stems and leaves (Figure 2A). The expression of LOC_Os03g40440 was only detected in the spikelets. For *OsB12D1*, the mRNA level achieved the peak in the seed embryo at 24 h after imbibition (HAI). In addition to the germinating seeds, *OsB12D1* was also highly expressed in root. LOC_Os07g41340, the tandem duplicate of *OsB12D1*, obtained similar expression style. Generally consistent with RT-PCR results, the digital expression patterns indicated the OsB12Ds were highly expressed in germinating seed and low in dry seeds, stems and leaves (Figure 2B). The two pairs of tandem duplicates had similar expression patterns, while the LOC_Os07g17330 and *OsB12D1* were the major transcripts with a higher expression level over their corresponding tandem duplicates. Differing from the RT-PCR result, LOC_Os07g17330 was also detected highly expressed in root (Figure 2B).

To obtain the gene regulation information about *OsB12D*s, gene responses to different factors that influence seed germination were detected. Those factors were phytohormones GA_3_ and ABA, osmotic reagent mannitol, salt, temperature, H_2_O_2_, respiration inhibitors NaN_3_ and SHAM (salicyl-hydroxamic acid), and flooding. Except for being significantly inhibited by mannitol and insensitive to the low concentration of H_2_O_2_ uniformly, four *OsB12Ds* were differentially responsive to the other factors. GA_3_ and high temperature pre-treatment could promote seed germination and impose positive effects on the *OsB12D* expression to some extent. However, although the NaCl and NaN_3_ entailed remarkably germination viability decline, some *OsB12D*s still could be induced by them, especially for Os07g17330. *OsB12D1* was not significantly induced by any detected factors; it was inhibited by ABA, mannitol, SHAM, high concentration of H_2_O_2_ and flooding, and insensitive to the other factors (Figure 2C,D). Similar responses occurred on its tandem duplicate LOC_Os07g41340.

*Cis-*elements analysis was performed based on the 1 kb promoter sequence upstream of the translation start point of the *OsB12D*s (Table S1). The pOs07g17330 had various *cis*-elements including stress responsiveness G-box, ABRE, GARE, TCA-element and GC-motif, which indicated LOC_Os07g17330 could respond to multiple germinating impact factors, this is consistent with the RT-PCR result. Unlike *HvB12Dg1*, *OsB12D1* harbored no GA response element within the promoter, which indicated it might not be regulated by GA. In addition to the G-box and ABRE, *OsB12D1* also harbored the anaerobic induction element within the promoter region, which implied *OsB12D1* could be induced by the anaerobia.

### 2.3. OsB12D1 Positively Responds to the Anoxia, Submergence and Flooding

**Figure 3:**
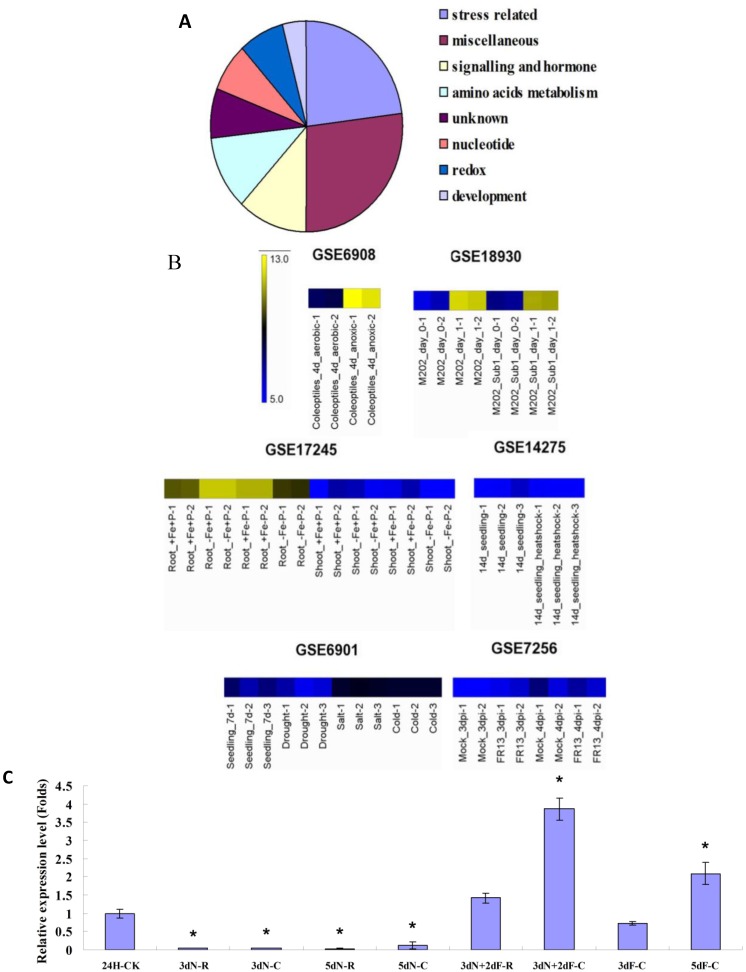
Stress response analyses of *OsB12D1*. (**A**) Functional categorization of the OsB12D1 coexpression proteins, the detailed coexpression information is presented in the Table S2; (**B**) Heat maps of *OsB12D1* response to different stresses. *OsB12D1* was insensitive to the heat shock (GSE14275), drought, salt, cold (GSE6901) and *Magnaporthe grisea* (GSE7256), but was induced by anoxia (GSE 6908), submergence (GSE18930) and Fe deficiency (GSE17245). Data were obtained from the Rice Oligonucleotide Array Database [25]; (**C**) qRT-PCR analysis of the *OsB12D1* expression in response to flooding stress. The dehulled seeds were germinated in normal conditions “N” for three days, and then switched to the flooded condition “F” for two days; the coleoptiles and roots were collected for RNA extraction. The coleoptiles and roots from three- or five days of normal growth and flooding-stressed seedlings also were collected. Embryos from 24 HAI seeds, 24H-CK, under normal conditions were used as internal reference; 3dN-R/-C or 5dN-R/-C, roots “R” or coleoptiles “C” of 3 or 5 days germinating seeds under normal condition; 3dN+2dF-R/-C, roots or coleoptiles of seedlings switched from 3 days normal germination to flooding for two days; 3dF-C or 5dF-C, coleoptiles from 3 or 5 days germinating seeds under flooding. The expression of *OsB12D1* was normalized to the expression of the rice 18S rRNA. The values are the means with the standard deviations (*n* = 3), asterisks (* *p* < 0.01, Student’s *t*-test) represent significant differences from the 24 H-CK expression level.

The potential genes that are coexpressed with *OsB12D1* were searched on the ricearray database [25]. Many biotic or abiotic stress-related genes were obtained; including some stress-related, miscellaneous, redox, transport, amino acids metabolism and signaling-related genes (Figure 3A). Notably, four anoxic or anaerobic stress-related proteins were included, that are ethylene-responsive element-binding protein (EREBP-1), alcohol dehydrogenase (ADH) and two non-symbiotic hemoglobin 2 proteins (rHb2), which suggested that *OsB12D1* might respond to the anoxic or anaerobic stress.

To obtain the general view of *OsB12D1* response to stress, the ricearray results related to various stress conditions were retrieved [25]. These stress conditions included: subjected 14-day-old light-grown rice seedlings to 42 °C heat shock for 10 h (GSE14275); subjected seven-day-old light-grown rice seedlings to drought, salt and cold stress for 3 h (GSE6901); treated two-week-old rice plants with *Magnaporthe grisea* (virulent isolate FR13) (GSE7256); rice seed germinated under oxygen-free (anoxia) environment in the dark at 28 °C for four days (GSE 6908); submerged 14-day-old rice plants of submergence tolerant M202(*Sub1*) and intolerant M202 for 24 h (GSE18930). The results showed that the OsB12D1 was insensitive to the heat shock, drought, salt, cold and *Magnaporthe grisea* infection, but was significantly induced in the anoxic coleoptiles and submerged seedlings [26], which implied that OsB12D1 might function in anoxia and submergence resistance (Figure 3B).

While in the seed embryos of 24 HAI under flooding, the expression of *OsB12D1* was very low (Figure 2A). To further check the *OsB12D1* response to the submergence, its expression under flooding during the seed germination and early seedling growth was detected. After germinating under normal conditions for three days when the visible radicles emerged, the rice seedlings were transferred to grow under the flooded condition for two days, and then the coleoptiles and roots (not including hypocotyl) were collected to evaluate the mRNA accumulation of *OsB12D1*. A lower *OsB12D1* expression level was detected in the coleoptiles and roots under normal conditions. After two days of flooding, the expression of *OsB12D1* was increased greatly both in the coleoptiles and roots, especially in the coleoptiles (Figure 3C). Meanwhile, although there was no radicle protrusion, the *OsB12D1* could be induced greatly in the coleoptiles of the three or five-day-old seedlings under sequential flooding (Figure 3C). These results confirmed that the *OsB12D1* is a flooding-responsive gene.

### 2.4. Over Expressing of OsB12D1 Enhances Rice Flooding Tolerance during Seed Germination and Early Seedling Growth

**Figure 4:**
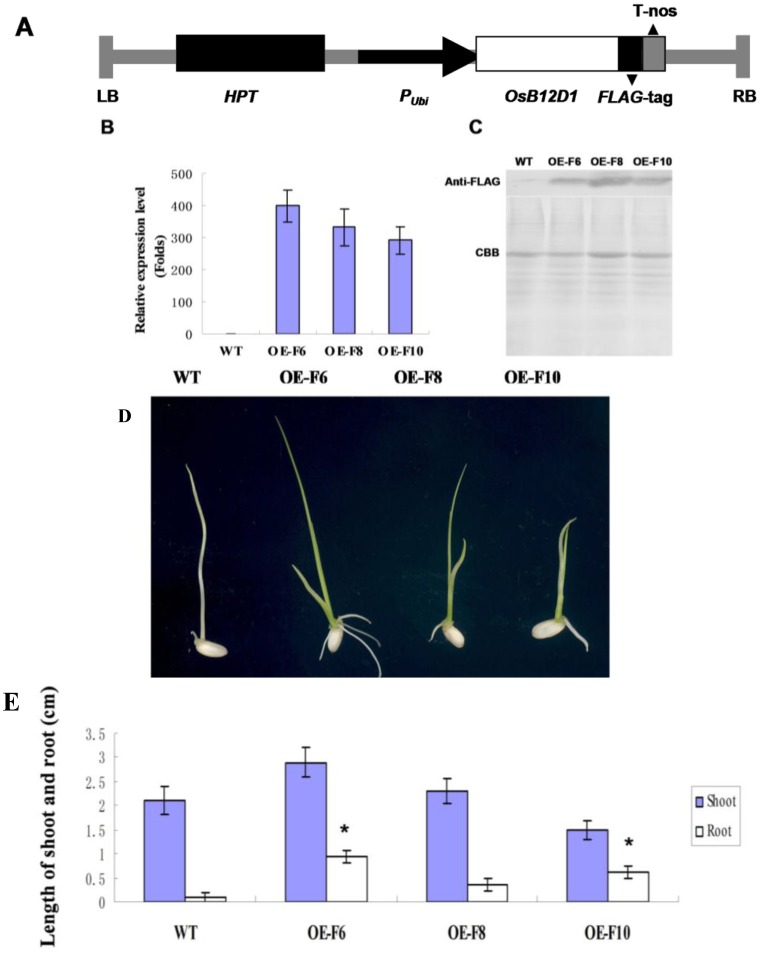
Overexpressing of *OsB12D1* enhanced rice seed flooding tolerance. (**A**) Schematic diagram of the *pUbi-OsB12D1*-*FLAG* construct used for transformation. LB, left border; HPT, hygromycin B phosphotransferase; Pubi, maize ubiquitin promoter; *OsB12D1*, *OsB12D1* cDNA; *T-nos*, nos terminator; RB, right border; (**B**,**C**) qRT-PCR and western blot analyses of *OsB12D1* overexpression in transgenic lines. WT, Zhonghua11; OE-F6, OE-F8, and OE-F10, three individual transgenic lines; CBB R250-stained gel served as a loading control; (**D**,**E**) *OsB12D1*-OE seeds exhibit flooding tolerance during germination. (**D**) *OsB12D1*-OE seeds were submerged in water 8 cm deep for seven days. Three independent experiments were performed; 8–10 seeds were used for each replicate; (**E**) the length of shoot (coleoptile) and root of the *OsB12D1*-OE seedlings under flooding for seven days; asterisks (* *p* < 0.01, Student’s *t*-test) represent significant different from wild type Zhonghua 11.

To investigate the function of *OsB12D1*, an over expression construct of *pUbi*-*OsB12D1-FLAG* was transformed into rice cultivar Zhonghua11 (Figure 4A). Ten independent positive T0 transformants were confirmed by genomic DNA PCR with primer pair pUbi/OsB12D1-R. Three transgenic lines of the T2 generation with the high expression level of *OsB12D1* were used for further study. The gene over expression was detected at the mRNA and protein level, and the transcripts were accumulated more than 300-fold higher than that in the wild type (Figure 4B,C).

The seed of all three *OsB12D1*-OE transgenic lines germinated well under normal growth conditions; there was no significant difference from the wild type (Figure S1), and no visible phenotypes were found throughout the whole plant’s life.

Considering the above analyses, *OsB12D1*-OE seedlings response to flooding was investigated. The dehulled seeds of *OsB12D1*-OE lines and the wild type Zhonghua11 were submerged in 8 cm of distilled water and grown under normal conditions. In the first three days, the coleoptiles of all seeds were elongated and no significant differences were observed between the mutant and wild type. After five days of submergence, visible radicles appeared in the *OsB12D1*-OE seeds. After seven days, all three *OsB12D1*-OE seeds protruded radicles successfully, although they had cracked coleoptiles and outgrew their primary leaves. Notably, line OE-F6 could extend both of the seed roots and adventitious roots under the flooded condition (Figure 4D,E). When the submergence time was prolonged to 10 days, the root could grow quickly, and the transgenic seedling could grow continuously and emerge from the water. However, for the wild type, no radicle protruded and the coleoptiles would bend as the submergence time was prolonged (Figure S2). Upon being transferred to the mud, all *OsB12D1*-OE seedlings could survive and grow better than the wild type.

GA and ethylene are two important regulators for plant flooding escaping [27]. The seedling phenotype of OsB12D1-OE lines under flooding containing GA and ethylene biosynthesis inhibitors were detected. The GA biosynthesis inhibitor paclobutrazol (PBZ) retarded seed germination under flooding, while after five days of submergence, the visible radicles emerged in the mutant seedlings but not in the wild type (Figure S3), which suggested that OsB12D1-OE seeds could enhance radicle protrusion without GA biosynthesis under flooding. Ethylene biosynthesis inhibitors CoCl_2_ could retard the coleoptiles elongation, as well. After five days of submergence with CoCl_2_, all seedlings including the wild type could be observed with radicle protrusion, while there was no significant difference between the wild type and the mutant, thus implying that OsB12D1 could promote radicle protrusion under flooding in correlation to the ethylene.

### 2.5. OsB12D1 Is Mainly Located in the Mitochondrion and Might Enhance Electron Transfer

**Figure 5:**
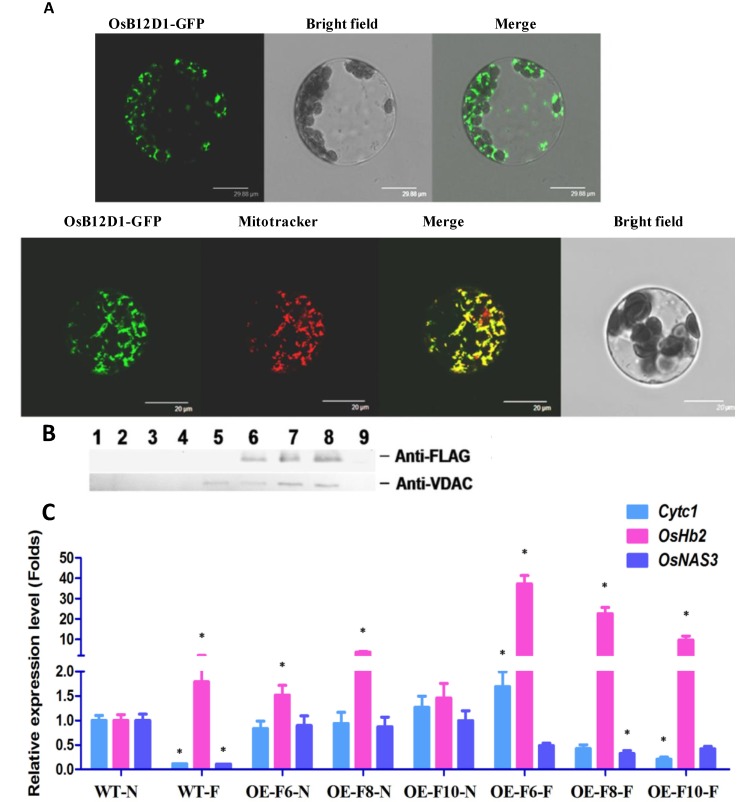
Subcelluar localization of OsB12D1 and the expressional patterns of electron transfer-related genes. (**A**) Transient expression of *OsB12D1-gfp* in the *Arabidopsis* leaf protoplast. OsB12D1-GFP, green fluorescence of OsB12D1-GFP under a dark field; Bright field, morphology of chloroplasts under a bright field; Mitotracker, red fluorescence of mitochondria stained by MitoTracker Red (Molecular Probes); merge, merged images of green fluorescence with Bright field or MitoTracker. Bars =29.88 or 20 μm; (**B**) Western blot analysis displayed the OsB12D1-FLAG protein enriched in the mitochondria fractions of the percoll gradient. After ultracentrifugation for mitochondria isolation, the percoll gradient was fractionated into nine 1 mL fractions from top to bottom, and used for Western blot with the first antibody of anti-FLAG and anti-VDAC (antibody of mitochondrial mark protein); (**C**) qRT-PCR analyses of the expression of *Cytc1*, *OsHb2* and *OsNAS3* in the seedlings of *OsB12D1*-OE lines. The *OsB12D1*-OE seeds were germinated under normal “N” and flooding condition “F” for three days, and then the coleoptiles were collected for qRT-PCR analyses. The expression of *OsB12D1* was normalized to the expression of the rice 18S rRNA. The values are the means with the standard deviations (*n* = 3), asterisks (* *p* < 0.01, Student’s *t*-test) represent significant different from WT-N expression level.

According to the Pfam prediction, B12D might be a mitochondrion-located protein [28]. To validate this prediction, a construct of p35S*-OsB12D1-gfp* was introduced into the transient expression system of the *Arabidopsis* protoplast to investigate the subcellular localization of OsB12D1. The florescence signal of OsB12D1-GFP was observed to well match the RFP signal of MitoTracker Red but not overlap the chlorophyll (Figure 5A), which indicated that the OsB12D1 is mainly located in the mitochondrion.

To confirm the transient expression result, Western blot analyses were performed on the purified mitochondria. Firstly, the OE-F8 seeds were germinated under about 1 cm water in the dark for seven days to obtain etiolated *OsB12D1-FLAG*-OE seedling for mitochondrial isolation. Consistent with the result of flooding stress, OE-F8 seeds extended the seed roots and adventitious roots more rapidly than the control under waterlogging. Western blot results showed that the highest signal of anti-FLAG and anti-VDAC coexisted in the seventh and eight lanes where the mitochondria-enriched fractions are located, thereby supporting the transient expression result (Figure 5B).

From the Pfam annotation, B12D is a potential constituent of NADH–ubiquinone reductase of the electron transport chain complex I [28]. Except for response to the submergence and anoxia, the *OsB12D1* was also found to be induced by iron (Fe) deficiency (Figure 3B; GSE17425; [29]). Fe is important for mitochondrial electron transport since all related enzymes contain Fe. Meanwhile, OsB12D1 is highly coexpressed with two non-symbiotic hemoglobin-2 proteins (Hb2; correlation coefficient are 0.7084 and 0.6848), the Fe and oxygen binding proteins. All those observations suggested that *OsB12D1* might be involved in mitochondrial electron transport. To validate this hypothesis, we selected three related genes to detect their expression in the germinating seeds of *OsB12D1*-OE lines under normal and flooding conditions. Those genes are the mitochondrial electron transport-related gene *Cytc1*, the non-symbiotic hemoglobin-2 gene *OsHb2,* and the Fe transport-related gene *OsNAS3*. For the wild type, *Cytc1* and *OsNAS3* were significantly repressed by flooding, whereas in the *OsB12D1*-OE lines, the repressions were not remarkable, especially for OE-F6, the *Cytc1* was increased. *OsHb2* had relatively high expression in the three *OsB12D1*-OE lines over the wild type under normal conditions (Figure 5C). Under flooding, the *OsHb2* could be induced in various lines, and the expression level in OE-F6 was up to 37 times higher than that in the wild type (Figure 5C). These results suggested that OsB12D1 might enhance the electron transfer through coordinating the expression of related genes.

## 3. Discussion

The phylogenetic analyses revealed that the amino acid sequence characteristic of OsB12D1 protein is highly conserved from moss to angiosperms. In rice, six *OsB12D*s shared similar gene sizes and protein sequences, while having different exon numbers and intron sizes. This indicated that the long evolutionary history entailed huge variation on the gene structure, which might silence or change the gene function [30]. The two pairs of tandem duplicates shared a similar expression pattern; LOC_Os07g17330 and LOC_Os07g41350 were the major transcripts. For lower expression level, LOC_Os07g17310 and LOC_Os07g41340 would tend to lose its function in the future [31]. Similar phenomena existed in barley, at least eight to nine B12D copies had been detected by primer extending experiments, while only one or two major transcripts presented in all detected tissues [23]. As a *Balem*-like gene, *OsB12D*s were mainly expressed in the germinating seed and developing seed [18], while they were also differentially presented in other tissues, especially in the root. RT-PCR showed both germination promoter and inhibitor could drive the transcription of certain *OsB12D*s. Differential expression pattern indicated that functional divergence of *OsB12D*s appears along with evolution, and the multiple gene copies remaining in the genome will favor the species environment adaptation. 

*Balem* genes can respond to various cereal seed germination signals [19,22,23]. The expression of the barley *Balem* gene HvB12Dg1 was suppressed by germinating inhibitor ABA and osmotic agent mannitol, but induced by GA in germinating seeds for carrying the gibberellic acid response complex (GARC)-like element in the promoter region [23,32], which implied the B12D might promote seed germination through a GA-dependent way. In this study, *OsB12D1* was greatly inhibited by ABA and mannitol, while weakly improved by GA_3_. None of the other eight detected factors that affected seed germination could induce the *OsB12D1* expression significantly. Meanwhile, overexpression of *OsB12D1* showed no significant difference in germination ability under normal conditions. Since *OsB12D1* was also highly expressed in the root, these observations suggested that *OsB12D1* could not only respond to some seed germination-related signals, but also was regulated by other factors.

The coexpression prediction showed that *OsB12D1* might participate in the stress response, especially for anoxic or anaerobic stress. The microarray data supported this prediction. *OsB12D1* was probed to be greatly induced in the four-day-old anoxic coleoptiles and in both of the tolerant or intolerant 14-day-old rice plants submerged for 24 h [26,33]. Consistently, the promoter of *OsB12D1* harbored two *cis*-elements of anaerobic induction motif. However, from the transcriptome data of germinating seeds, in the first 30 h under anaerobic stress, the *OsB12D1* was induced very slowly in the rice embryos, and the expression of *OsB12D1* was down-regulated when germinating seeds were switched from the aerobic to anaerobic conditions [9]. Anoxia or hypoxia is the primary signal triggered by flooding stress [34]. To understand the above seemingly contradictory results, we investigated the expression of *OsB12D1* in response to the flooding in the geminating seeds and seedlings. RT-PCR displayed that the expression level of *OsB12D1* was very low at the first 24 h of flooding, but increased significantly along with the prolonged flooding time (Figure 2D and Figure 3C), and the expression was induced both in the roots and coleoptiles when the three-day-old normally grown seedlings were transferred to flooded conditions for two days. This result also explained why *OsB12D1* was abundant in the root. Because rice is a semiaquatic crop, the roots are always submerged in mud. From the above results, it was deduced that *OsB12D1* was sensitive to oxygen deficiency, while at an early stage of seed germination, the gene expression could be delayed by anoxic or anaerobic stress.

Under flooded conditions, subjected to the stresses of hypoxia and mechanical damage, the rice seed failed to protrude the radicle during germination [14]. The phenotype of germinating seeds under multi-treatments showed that the radicle is sensitive to various stresses (Figure 2C). Radicle emergence is the physiological indicator of the seed germination [35]. In this study, *OsB12D1-*OE seed radicles protruded and there was also an establishment of adventitious roots under flooded conditions. Additionally, the transgenic seedlings grew continuously and emerged from the water. After transplanting, the *OsB12D1-*OE seedlings grew better than the control with roots to adapt to the environment. The roots of the *OsB12D1*-OE etiolated seedling grew more quickly than the control under waterlogging, too. These results confirmed that the *OsB12D1* can enhance flooding or waterlogging tolerance during seed germination and early seedling growth. The direct crop seeding in farming practice is low cost and simple in comparison to transplanting. However, its large-scale adoption is restrained. One of the major obstacles for rice direct seeding is poor seedling establishment by waterlogging or flooding stress in the uneven land [36]. Therefore, fine regulation of the expression of *OsB12D1* could contribute to the spread of direct seeding practices. Because of the high conservation of OsB12D1 sequence across plants, this effect of flooding tolerance possibly exists in other crops.

According to the Pfam annotation, B12D is analogous to the bovine mitochondrial MLRQ protein, a potential constituent of NADH-ubiquinone reductase of the electron transport chain complex I [37]. The subcellular localization results validated that OsB12D1 is mainly localized in mitochondria. During seed germination, along with the function recovery of electron transport chain, the ATP content increased at the first 24 HAI, and then decreased [38]. Although the qRT-PCR result showed that the transcript of *OsB12D1* under normal conditions had a similar accumulation style with ATP, however, without consensus phosphorylation sites and cysteine-rich motifs like MLRQ, OsB12D1 is unlikely to participate in the electron transport and ATP biosynthesis directly [39]. Under flooding, mitochondrial electron transport-related gene *Cytc1* and Fe transport-related gene *OsNAS3* still maintained certain expression activity in the *OsB12D1-*OE lines, while the Fe and oxygen binding-related gene *OsHb2* was significantly induced in the *OsB12D1-*OE lines. Nonsymbiotic hemoglobinsc can sequester oxygen under hypoxia and promote the NADH oxidization to provide ATP for cell growth and development [40]. In *Arabidopsis*, overexpression of *AHb2* can improve oxygen availability within developing seeds [41]. Therefore, OsB12D1 might enhance electron transport in mitochondrion through mediating Fe and oxygen availability, and then contribute to ATP biosynthesis under flooded conditions.

Rice is the only cereal plant that can germinate under flooded conditions through a rapid elongation of coleoptile. This process is inhibited by ABA but promoted by ethylene and GA, associated with carbohydrate consumption [15,27]. In this study, RT-PCR indicated that GA_3_ exerted a weak effect on the expression of *OsB12D*1, while the radicle protrusion of *OsB12D1*-OE lines under flooding was not affected by the GA inhibitor PBZ. Ethylene had been detected increasing quickly in the seedlings after three days of germination under flooding [15]; the *OsB12D1* transcript also had a similar accumulation style, and ethylene-responsive transcription factor coding gene AP2/EREBP was highly coexpressed with *OsB12D1* (Table S2; correlation coefficient is 0.662). Ethylene is a power inhibitor of the root growth [42]. In this study, the ethylene biosynthesis inhibitor CoCl_2_ exhibited consistent promoting effects on the radicle protrusion of the wild type and *OsB12D1*-OE lines under flooding. These results indicated that the *OsB12D1* gene might have a functional correlation with the ethylene while remaining independent of GA signaling.

## 4. Materials and Methods

### 4.1. Rice Seed Imbibition and Germinating Conditions

The dehulled rice (*Oryza sativa* L. japonica. cv. Nipponbare) seeds were washed with distilled water three times and then imbibed in distilled water under 26 °C, 100 mmol·m^−2^ s^−1^ white light with a 16 h light/8 h dark cycle. The embryos of seeds were collected at intervals of 0, 24, 48 and 72 h after imbibition, respectively. For multiple germination factors’ treatment analyses, the seeds were treated at 11 different conditions as follows: imbibed in water incorporated with 200 µM GA_3_, 50 mM ABA, 200 mM NaCl, 20 mM H_2_O_2_, 1 mM NaN_3_, 20% Mannitol and 15 mM SHAM, respectively; flooding with seeds directly submerged in 8 cm deep distilled water, and high- or low-temperature treatment with seeds pretreated at 40 °C and 100% relative humidity or 4 °C imbibition in the dark for three days before being transferred to germinate in benign conditions. After 24 h of imbibition, the embryos of treated seeds were sliced for RNA extraction. For an overexpression test, the mutant seeds were imbibed for 24 h and the embryos collected to extract RNA and protein for RT-PCR and Western blot analysis. For the flooding response experiment, the seeds were geminated at normal conditions for three days, and then switched to the flooded conditions for two days; subsequently, the coleoptiles and roots were collected for RNA extraction. For GA or ethylene biosynthesis inhibitor response detection, the seeds were germinated in 8 cm of water incorporated with 200 µM paclobutrazol (PBZ) and 60 µM CoCl_2_, respectively.

### 4.2. RNA Extraction and Quantitative Real-Time PCR

Total RNA was extracted using Trizol reagent (TOYOBO, Kyoto, Japan) from the above samples and 60-day-old rice plant tissues including root, leaf, stem and spikelets. The quality of RNA was checked by agarose gel electrophoresis. High-quality total RNA (1 μg) treated with DNase I (Fermentas, Waltham, MA, USA) was reverse-transcribed using a First Strand cDNA synthesis kit (ReverTra Ace, TOYOBO, Kyoto, Japan), 10-fold diluted cDNA samples were used for qRT-PCR with SYBR Green Real-time PCR Mix (Bio-Rad, Hercules, CA, USA), two biological and three technical replicates analysis were performed on CFX96 Real-time system (Bio-Rad, Hercules, CA, USA). PCR amplification programs was: 95 °C for 3 min, then 40 cycles of 95 °C for 10 s, 60 °C for 15 s, and 72 °C for 15 s, followed by 72 °C for 10 min. The mRNA levels were normalized to the amplication of the rice 18S rRNA according to the 2^−ΔΔt^ method [43]. The relative expression level was referred to the mRNA level in 24 HAI seed embryos under normal conditions. Special primers were designed with the sequence information from the RGAP database (Table S3).

### 4.3. Generation of OsB12D1 Transgenic Rice

The full-length cDNA of *OsB12D1* was amplified using primer pair OE-F/OE-R and inserted into the modified PUC1301 vector, resulting in an *OsB12D1* overexpression vector fused with FLAG-tag driven by the maize ubiquitin promoter. The construct was transformed into *Oryza sativa* cv. Zhonghua 11 using the *Agrobacterium tumefaciens*-mediated method [44]. All seeds were harvested from plants grown simultaneously in the greenhouse of Wuhan Botanic Garden and then stored for four weeks before usage.

### 4.4. Protoplast Transient Expression Assay

The full-length cDNA of *OsB12D1* was amplified using primer pair Sub-F/Sub-R and inserted into vector pM999GFP (kindly provided by Dr. Jian Xu, Huazhong Agricultural University, China), resulting in a *C*-terminal fusion with GFP driven by CaMV 35S promoter. The transient expression in *Arabidopsis* protoplasts was performed by PEG/calcium-mediated transformation [45]. Fluorescent signals were examined at 24 h after transformation using confocal laser microscope (Lecia SP2, Dresden, Germany). GFP green images were obtained at 488 nm argon laser. Mitochondria were stained with MitoTracker Red (Invitrogen, Carlsbad, CA, USA) according to the manufacturer’s protocol. The red fluorescence was obtained at 543 nm HeNe laser. All subcellular analyses were performed at least three times in independent experiments.

### 4.5. Mitochondrial Isolation

The Percoll gradient method was used for mitochondrial isolation from *OSB12D1-FLAG-OE* etiolated seedling [46]. After ultracentrifugation, the Percoll gradient was fractionated into nine 1 mL fractions from top to bottom, after being diluted with washing buffer five times, and the fractions were spun at 22,000 *g* for 15 min at 4 °C to collect the pellets. The pellets were then dissolved with loading buffer and used for Western blot analysis.

### 4.6. Western Blot

Proteins were separated by 15% SDS-PAGE and electro-blotted onto PVDF. The blot was probed with Anti-FLAG (Sigma, San Diego, CA, USA) or anti-VDAC (Sigma; mitochondrial membrane voltage-dependent anion channel, a mitochondrial mark protein) followed by alkaline phosphatase conjugated secondary antibody (Sigma, San Diego, CA, USA).

### 4.7. Database Screening and Bioinformatics Analyses

Homologs of the OsB12D1 protein sequence in different species were searched using the BLASTP program of NCBI. For rice-conserved homologs, the key word ‘‘B12D’’ was used as query to search against the MSU-RGAP Database. Multiple alignments were performed by MUSCLE software [47]. MEGA software (version 4.0) was used to perform the phylogenetic analysis. The coexpression prediction, stress microarray data and heat map of the anatomical expression patterns of *OsB12D* genes in different tissues/organs were analyzed on the Rice Oligonucleotide Array Database [25]. Gene structure was analyzed by GSDS [48]. The sequence conservation of amino acids was analyzed by DNAMAN software [49]. The conserved motifs and transmembrane domains were annotated according to MEME [50] and TMHMM [24], respectively.

## 5. Conclusions

In this study, we firstly uncovered the evolution, structure, expressional pattern, function, and protein subcellular localization of a new *Balem*-like gene *OsB12D1* systematically. *OsB12D1* belongs to an ancient gene family and was mainly expressed in the germinating rice seed and root. *OsB12D1* could be significantly induced by flooding stress, and overexpression of *OsB12D1* could enhance rice flooding tolerance during seed germination by promoting seedling establishment. OsB12D1 is mainly located in the mitochondrion and might be involved in Fe and oxygen availability under flooded condition. The function of *OsB12D1* could be regulated by a variety of signal factors, including oxygen, ABA, ethylene, and Fe deficiency. How can it be regulated by orchestra signals under flooding and contribute to the radicle protrusion? The interaction protein or gene regulatory factor isolation will help to resolve these questions in the future.

## Supplementary Files

Supplementary File 1
