# Supplementary material for: The Mitochondrion-Located Protein OsB12D1 Enhances Flooding Tolerance during Seed Germination and Early Seedling Growth in Rice
Source: Int J Mol Sci. 2014 Jul 31;15(8):13461–81. doi: 10.3390/ijms150813461 (PMC4159805; doi:10.3390/ijms150813461)

Supplementary Information

Supplemental Dataset 1. Multiple alignments of amino acid sequences of OsB12D1 and its closest homologs from 29 different plant species by MUSCLE software. (see the attached Supplemental Dataset 1 txt.)

Table S1. *Cis*-elements analysis based on the 1 kb promoters sequence upstream of the translation start point of the *OsB12D*s using software of Place.

Site Name	Sequence	Function	Position within the Gene Promoter					
			**Os03g40440**	**Os06g13680**	**Os07g17310**	**Os07g17330**	**Os07g41340**	**OsB12D1**
ABRE	ACGTG	abscisic acid responsiveness	(−)316 (+)326			(−)784	(+)356	(+)48 (−)221
ABRE	MACGYGB		(−)71	(+)165	(+)192 (−)231	(+)855 (+)865 (+)991		(−)194
motif IIb	CCGCCGCGCT		(−)344					
P-box	CCTTTTG	gibberellin-responsive element		(−)722		(−)525		
P-box	TTTTTTCC						(−)271	
GARE-motif	(T)AACAGA					(−)720		
TCA-element	CCATCTTTTT	salicylic acid responsiveness			(+)424 (−)783	(−)211 (−)236		
GC-motif	CCCCCG	anoxic specific inducibility		(−)318 (−)416 (−)370		(+)739 (−)937		
G-box	CACGTG	Stress responsiveness				(+)785		(+)195
ARE	TGGTTT	anaerobic induction		(+)786		(+)479	(+)671 (−)874 (+)984	(−)303 (+)870

Table S2. Coexpression analysis of *OsB12D1* on the website of Rice Oligonucleotide Array Database.

ID	Positive Coexpression	Binco	BinName
loc_os07g41340	0.7616	33.99	Development unspecified
loc_os03g12510	0.7084	21.3	Redox heme
loc_os02g39620	0.7077	20.2	Stress abiotic unspecified
loc_os02g39620	0.7077	20	Stress
loc_os01g67030	0.6991	17.2	Hormone metabolism auxin Induced regulated responsive activated
loc_os06g49340	0.6936	35.2	Unknown
loc_os03g13140	0.6848	21.3	Redox. Heme
loc_os01g67010	0.6848	17.2	Hormone metabolism auxin Induced regulated responsive activated
loc_os03g08460	0.662	27.3	RNA regulation of transcription.AP2/EREBP, APETALA2/Ethylene-responsive element binding protein family
loc_os12g38770	0.6604	26.13	Misc. Acid and other phosphatase
loc_os06g09870	0.6591	35.2	Unknown
loc_os03g25340	0.6551	26.12	Misc. peroxidase
loc_os11g47550	0.6496	20.1	Stress biotic
loc_os07g09630	0.6479	26.7	Misc. oxidases—copper, flavone
loc_os09g29480	0.645	13.1	Amino acid metabolism synthesis serine-glycine- cysteine group cysteine
loc_os08g40690	0.6449	20.1	Stress biotic
loc_os02g55890	0.642	34.3	Transport amino acids
loc_os03g45250	0.6357	13.1	Amino acid metabolism synthesis serine-glycine-cysteine group cysteine
loc_os12g09540	0.6343	23.1	Nucleotide metabolism synthesis purine GAR Synthetase
loc_os11g10510	0.6339	26.11	Misc. alcohol dehydrogenases
loc_os11g47560	0.6337	20.1	Stress biotic
loc_os01g20980	0.6331	10.8	Cell wall pectin esterases PME
loc_os09g31040	0.6177	30.3	Signaling calcium
loc_os11g47610	0.6154	20.1	Stress biotic
loc_os03g25280	0.6128	26.12	Misc. Peroxidases
loc_os05g48040	0.6118	34.99	Transport misc
loc_os03g49524	0.6087	26.2	Misc UDP glucosyl and glucoronyl transferases

Table S3. Primers used in this study.

Primers	Sequence (5'–3')
LOC_Os03g40440-F	GTTGGCATCGGGCATGGTGT
LOC_Os03g40440-R	GGAGAAGAAGCGGTTGATGG
LOC_Os06g13680-F	CAGCTCTTCCGCAACATCAC
LOC_Os06g13680-R	TGATCTCAGGGGTCTTGTCG
LOC_Os07g17310-F	TGAGGTGTACCCGCTGTTCG
LOC_Os07g17310-R	CAGAACCTCCTCACGCCAT
LOC_Os07g17330-F	AGGTGTACCCGCTCTTCCTC
LOC_Os07g17330-R	GAACTTCCTGAAGCCGTGCT
LOC_Os07g41340-F	CCGCCATGACCTTCGTCAC
LOC_Os07g41340-R	TGGTACTTCTCACCCTCCTC
OsB12D1-F	GTGGGAGGGATGTGCGTGTT
OsB12D1-R	TCGGAAGGCGTGGTGGTGAT

Table S3. *Cont.*

Primers	Sequence (5'–3')
18S-F	ACGAACAACTGCGAAAGC
18S-R	CGGCATCGTTTATGGTTG
Sub-F	ATCTAGAATGGGCCGTTGGGTGAAGCCA (*Xba*I)
Sub-R	CCTCTAGAGTTTTGACGGTCACTCTTGG (*Xba*I)
OE-F	AAGGTACCATGGGCCGTTGGGTGAAG (*BamHI*)
OE-R	AGGGATCCGTTTTGACGGTCACTCTTG (*EcoRI*)
Cytc1-F:	CGACCGTTTCCCACAACCCT
Cytc1-R:	CATGGCTATGGCACCACCAGG
OsHb2-F:	ACGAGGTTCGCGCTGCTTGA
OsHb2-R:	GGCTTCATCTCCTGCTTGATGG
OsNAS3-F:	CTGTTCACGGAGCTGGTGATG
OsNAS3-R:	GGCGAGCTGGATGTAGTTGTTG

Figure S1. Seeds germination of *OsB12D1-OE* lines under normal condition.

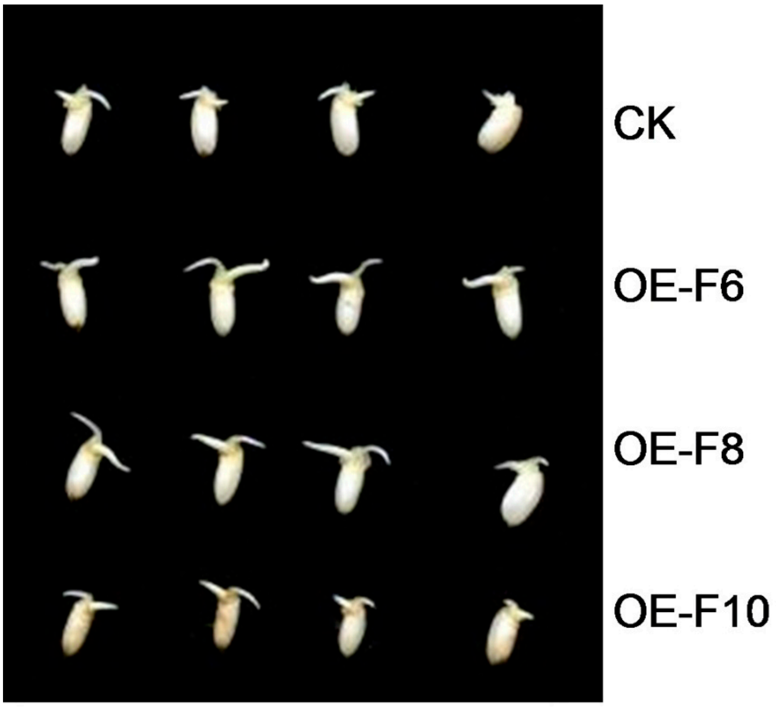

Figure S2. Ten-days-old seedlings of *OsB12D1-OE* lines under flooding**.** The arrows indicated the leaves emergency from the 8 cm deep of water. WT, Zhonghua11; OE-F6 and OE-F8, transgenic lines.

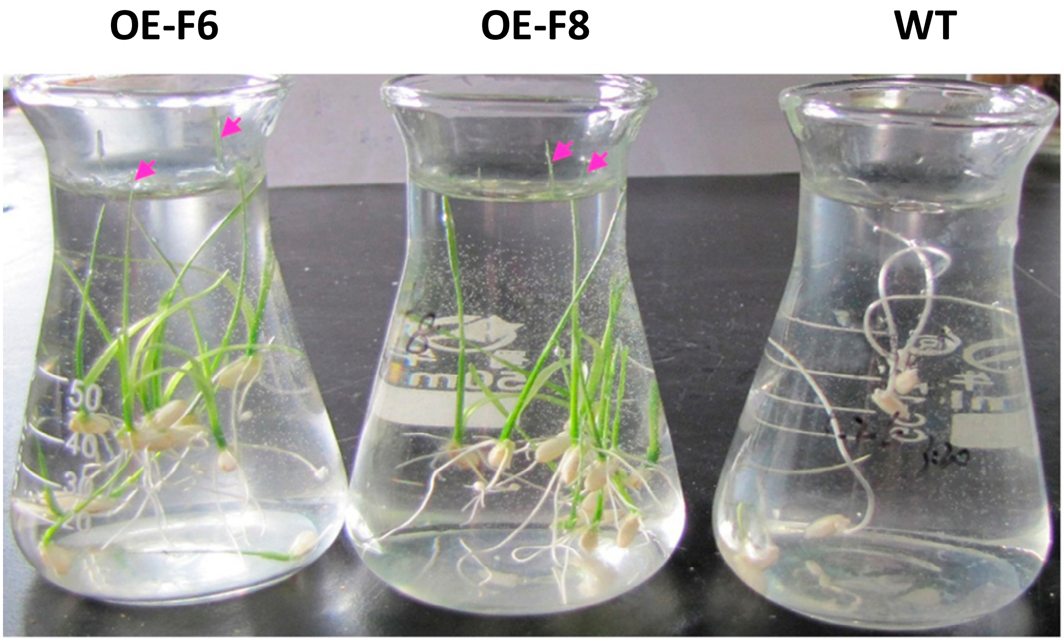

Figure S3. Five-days-old seedlings of *OsB12D1-OE* lines under flooding containing GA and ethylene biosynthesis inhibitors. PBZ, 200uM paclobutrazol, the GA biosynthesis inhibitor; $CoCl_2$, 60uM $CoCl_2$, ethylene biosynthesis inhibitor; WT, Zhonghua11; OE-F6 and OE-F8, transgenic lines.

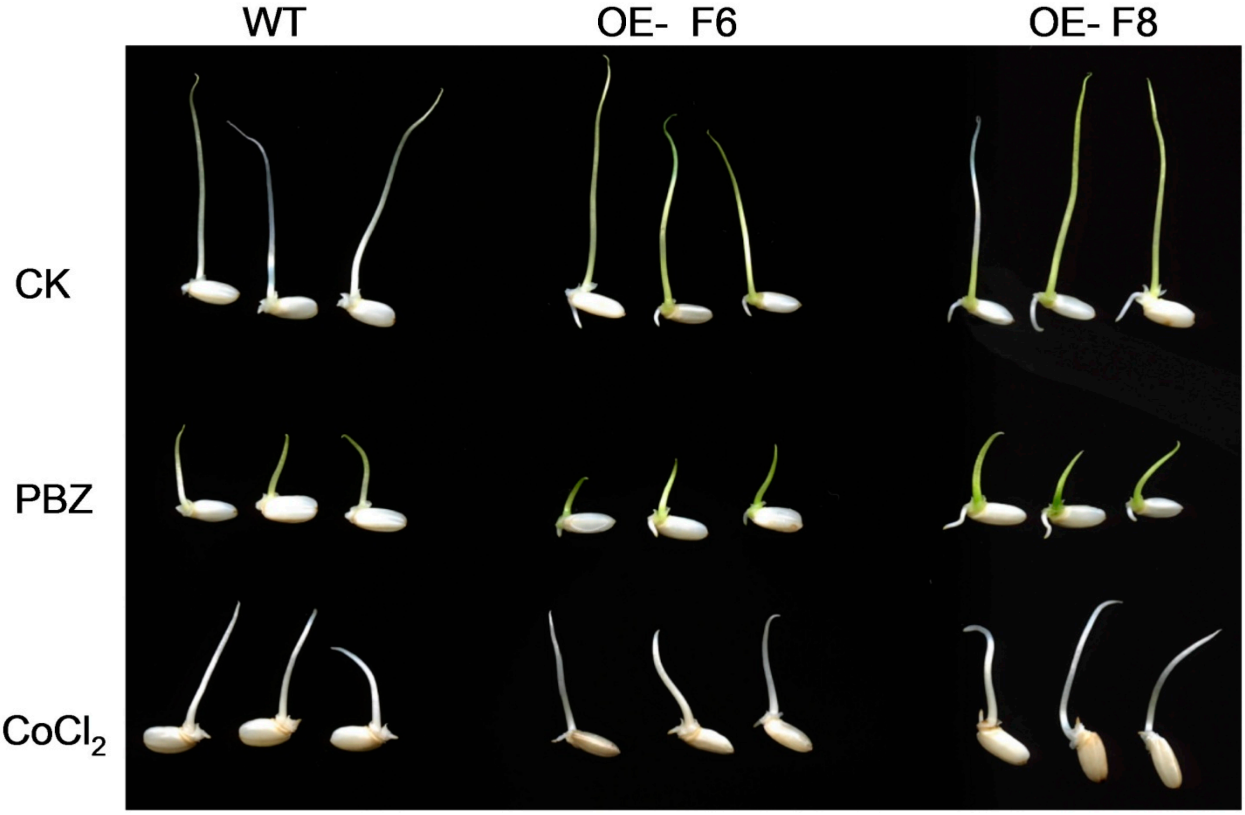

Supplement: Supplementary File 1 [file ijms-15-13461-s001.zip › ijms-57855-Supplemental final.pdf]
